# Sodium thiosulfate-catalysed synthesis of thioethers from aldehydes or carboxylic acids

**DOI:** 10.1098/rsos.250348

**Published:** 2025-10-01

**Authors:** Maral Salehi, Najmeh Nowrouzi, Mohammad Abbasi

**Affiliations:** ^1^ Department of Chemistry, Persian Gulf University, Bandar Bushehr, Bushehr Province, Iran

**Keywords:** sodium thiosulfate, thioether, aldehyde, carboxylic acid

## Abstract

This article presents a straightforward and efficient method for the synthesis of thioethers from the reaction of aldehydes or carboxylic acids with thiols, utilizing sodium thiosulfate as a catalyst and N,N-dimethylformamide (DMF) as a solvent. The coupling reactions, facilitated by sodium thiosulfate, lead to the formation of thioethers through the generation of stable thiyl radicals. These metal-free processes are highly valuable for constructing C–S bonds from readily available coupling partners. Various aldehydes and carboxylic acids, including 2-phenylpropionaldehyde, 2-phenylpropanoic acid, phenylacetic acid, 1-naphthaleneacetic acid and 2-furanacetic acid, were successfully employed as coupling partners. Notably, this method was also applied to structurally complex bioactive molecules, including the anti-inflammatory drugs indomethacin and ibuprofen, which contain a carboxylic acid group, successfully affording the corresponding thioethers in acceptable yields. The results demonstrate that sodium thiosulfate is an effective and practical catalyst for these transformations, offering a mild and environmentally benign strategy for C–S bond formation with broad substrate scope.

## 1. Introduction

Thioethers are essential compounds in both biological and industrial applications, and they feature prominently in fields ranging from pharmaceuticals [1] and agriculture to materials science and heterocyclic chemistry. The versatility and functionality of thioethers have driven extensive research into efficient and practical synthetic methodologies for C–S bond formation [2–5]. The alkylation of thiols is a foundational method in sulfide synthesis, typically involving reactions between thiols and alkyl halides in the presence of strong bases [6–10]. An alternative approach for thioether synthesis is the Mitsunobu reaction, where thiols couple with alcohols, offering a streamlined route to these compounds [11]. Transition-metal-catalysed C–S cross-coupling reactions between thiols and various electrophiles (such as organohalides, boronic acids and carboxylic acids) have also become core strategies in synthesizing thioether derivatives [12,13]. In the last two decades, sulfur-containing compounds such as sodium thiosulfate [14,15], sulfur [16], carbon disulfide [17–19] and thiourea [20–25] have emerged as flexible reagents for thioether synthesis, expanding available methodologies in this field. Direct thioetherification has also been advanced, with a notable approach using InBr_3_ and 1,1,3,3-tetramethyldisiloxane (TMDS) in a one-pot system to enable the efficient coupling of aromatic carboxylic acids and thiols. Similarly, a catalytic system of InI_3_ and TMDS has facilitated thioetherification of aliphatic carboxylic acids with thiols, providing a streamlined process for a range of substrates [26]. Another innovative synthesis pathway for selenide ethers with single or double C–Se bonds has been developed, using diselenides and phenylacetic acids. Under air-oxidative, metal-free conditions, compounds with one C–Se bond are synthesized, while a FeCl_3_/O_2_/Cs_2_CO_3_ system produces those with two C–Se bonds. Additionally, 1,2-diphenyldisulfane can be efficiently converted into sulfur ethers under these reaction conditions [27]. A decarboxylative thiolation protocol free from transition metals has also been reported, applicable to primary, secondary, tertiary (hetero)aryl acetates and *α*-CN-substituted acetates, yielding functionalized aryl alkyl sulfides in good to excellent yields. This method also accommodates C–Se bond formation from aryl diselenides, proving useful for the late-stage modification of pharmaceutical carboxylates with favourable selectivity and functional-group compatibility [28].

**Scheme 1.**
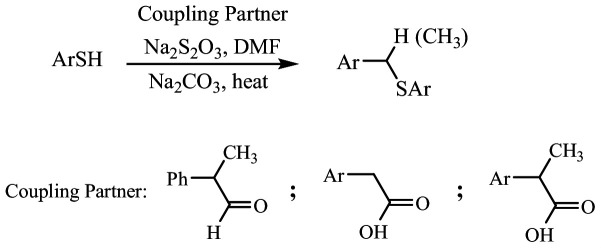
Synthesis of thioethers through the reaction of aldehydes or carboxylic acids with thiols.

Finally, a new direct synthesis method for aryl alkyl sulfides has been introduced using 2-phenylpropanal as a coupling partner. Diaryl disulfides react with this aldehyde in the presence of morpholine to produce sulfides in high yields. Additionally, disulfides generated *in situ* from aryl halides, CuI and cyanodithioformate can couple with 2-phenylpropanal to access aryl alkyl sulfides, expanding the synthetic approach available for sulfide construction [29]. Although the direct coupling of carboxylic acids or aldehydes with thiol sources via decarboxylation or decarbonylation process is important, the number of reported methods for this process remains very limited, to the best of our knowledge. This study introduces a straightforward and efficient method for synthesizing thioethers by coupling of aldehydes or carboxylic acids with thiols, facilitated by sodium thiosulfate as a catalyst in N,N-dimethylformamide (DMF; Scheme 1).

## 2. Results and discussion

We recently developed a simple method for synthesizing aryl alkyl sulfides through the coupling of 2-phenylpropanal and disulfides. This process involves sequential thioarylation and decarbonylation, performed in the presence of morpholine in DMF. Subsequently, we decided to expand this method by using a broader range of aldehydes and carboxylic acids as coupling partners with thiols in the presence of sodium thiosulfate as a catalyst.

To begin the study, we selected 2-phenylpropanal and thiophenol as the model starting materials. The reaction of 2-phenylpropanal (1.0 mmol) and thiophenol (1.0 mmol) in the presence of 20 mol% Na_2_S_2_O_3_ and K_2_CO_3_ (2.0 mmol) in DMF at 120°C for 24 h produced the desired sulfide product in 63% yield (table 1, entry 1). Encouraged by this result, we continued our study for possibility of further improvement in product yield. In the beginning, base screening was conducted. Although both K_3_PO_4_ and Bu_3_N afforded lower yields of product (table 1, entries 2, 3), the addition of 2.0 equivalents of Na_2_CO_3_ greatly improved the yield to 91%, after 6 h (table 1, entry 4). Reducing the amount of Na_2_CO_3_ to 1.5 mmol decreased the yield, but increasing it to 2.5 mmol had no effect on the results (table 1, entries 5, 6). The reaction did not proceed in the absence of base (table 1, entry 7). Compared with DMF, other solvents including DMSO, CH_3_CN, H_2_O and toluene were undesirable (table 1, entries 8−11). When the amount of Na_2_S_2_O_3_ was reduced to 10 mol%, the product yield dropped to 67%; however, increasing its amount to 30 mol% had no effect on the yield (table 1, entries 12, 13). A temperature of 120°C was the optimal temperature for this reaction. When the reaction temperature was lowered to 110°C, the yield also decreased. Higher temperature did not have any substantial impact on this transformation (table 1, entries 14, 15).

**Table 1.** Effect of different reaction parameters on the synthesis of thioethers^a^.

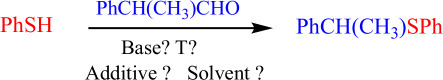						

entry	base (mmol)	catalyst (mol%)	solvent	T (^o^C)	time (h)	yield (%)
1	K_2_CO_3_ (2.0)	Na_2_S_2_O_3_ (20)	DMF	120	24	63
2	K_3_PO_4_ (2.0)	Na_2_S_2_O_3_ (20)	DMF	120	24	52
3	Bu_3_N (2.0)	Na_2_S_2_O_3_ (20)	DMF	120	24	40
4	Na_2_CO_3_ (2.0)	Na_2_S_2_O_3_ (20)	DMF	120	6	91
5	Na_2_CO_3_ (1.5)	Na_2_S_2_O_3_ (20)	DMF	120	6	60
6	Na_2_CO_3_ (2.5)	Na_2_S_2_O_3_ (20)	DMF	120	6	91
7	–	Na_2_S_2_O_3_ (20)	DMF	120	24	–
8	Na_2_CO_3_ (2.0)	Na_2_S_2_O_3_ (20)	DMSO	120	24	66
9	Na_2_CO_3_ (2.0)	Na_2_S_2_O_3_ (20)	CH_3_CN	reflux	24	–
10	Na_2_CO_3_ (2.0)	Na_2_S_2_O_3_ (20)	H_2_O	reflux	24	–
11	Na_2_CO_3_ (2.0)	Na_2_S_2_O_3_ (20)	toluene	reflux	24	51
12	Na_2_CO_3_ (2.0)	Na_2_S_2_O_3_ (10)	DMF	120	24	67
13	Na_2_CO_3_ (2.0)	Na_2_S_2_O_3_ (30)	DMF	120	6	90
14	Na_2_CO_3_ (2.0)	Na_2_S_2_O_3_ (20)	DMF	110	6	58
15	Na_2_CO_3_ (2.0)	Na_2_S_2_O_3_ (20)	DMF	130	6	92

^a^ isolated yields.

Having the optimized reaction conditions in hand, the substrate scope of the reaction was shown by the reaction of 2-phenylpropanal and different substituted thiols. The results of these investigations are presented in table 2.

**Table 2.** Synthesis of thioethers from the reaction of thiols and 2-phenylpropanal in the presence of sodium thiosulfate^a^^,^^b^.

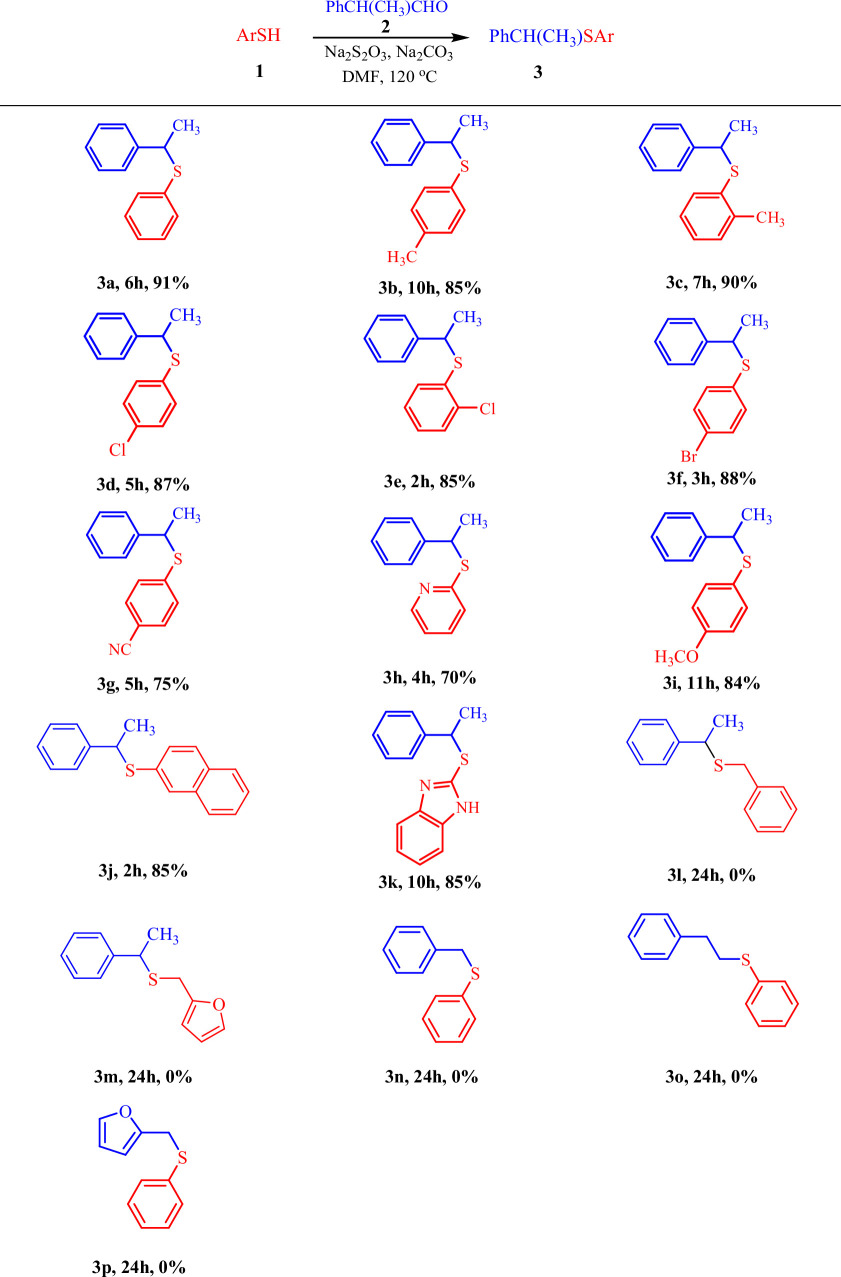			

^a^ Reaction conditions: thiol (1.0 mmol), aldehyde (1.0 mmol), Na_2_S_2_O_3_ (0.2 mmol), Na_2_CO_3_ (2.0 mmol) in DMF (1 ml) at 120°C.

^b^ The yields refer to the isolated pure products.

When aromatic thiols such as thiophenol, 4-methylthiophenol, 2-methylthiophenol, 4-chlorothiophenol, 2-chlorothiophenol, 4-bromothiophenol, 4-cyanothiophenol, 4-methoxythiophenol, pyridine-2-thiol, 1*H*-benzo[*d*]imidazole-2-thiol and naphthalene−2-thiol were used as thiol co-substrates, the corresponding sulfide products were obtained in high yields (table 2, compounds 3a–3k). The results indicate that aromatic thiols with electron-withdrawing substituents accelerate the reaction, allowing it to reach completion in a shorter time, although product yields remain consistent across both types of thiols. It appears that the reaction is not highly dependent on steric effects, and steric hindrance does not have a negative impact on the reaction progress. For example, the reaction of 2-methylthiophenol and 4-methylthiophenol produced the sulfide product with acceptable yields in nearly the same reaction time (table 2, compounds 3b and 3c). Despite the effective performance of aromatic thiols in these reactions, aliphatic thiols showed significantly lower reactivity. Benzyl mercaptan and 2-furfurylthiol did not yield the desired coupling product when reacted with 2-phenylpropanal, and only the corresponding disulfide was formed in these reactions (table 2, compounds 3l and 3m). This is probably due to the lower stability of the radical species of aliphatic thiols compared with aromatic thiols (RS. versus ArS.). To further extend the substrate scope, phenylacetaldehyde or 3-phenylpropanal was used instead of 2-phenylpropanal and reacted with thiophenol under the optimized conditions (table 2, compounds 3n and 3o). In these cases, no desired product was detected; instead, the corresponding disulfides were formed. 2-furanacetaldehyde, as a heteroaromatic aldehyde with an aliphatic linker, was also employed in the reaction; however, no product was formed in this case either (table 2, compound 3p). The failure of these aldehydes to participate in the reaction is probably due to the lower stability of their corresponding primary or less-stabilized radical intermediates, in contrast to the benzylic secondary radical formed from 2-phenylpropanal, which readily undergoes the transformation.

Following the successful application of sodium thiosulfate as a catalyst in the decarbonylative thioarylation of 2-phenylpropanal with thiols, we extended our study by replacing 2-phenylpropanal with 2-phenylpropanoic acid as the coupling partner. This allowed us to explore the synthesis of sulfides using the same catalytic system. When thiophenol was reacted with 2-phenylpropanoic acid, the corresponding sulfide was produced in 75% yield. Increasing the amount of Na_2_S_2_O_3_ or Na_2_CO_3_ had no effect on the yield, but increasing the reaction temperature to 140°C led to an improvement in yield (table 3, 5a, 88%). Therefore, the same optimal conditions from table 1 were applied for acids, with only the temperature increased. Under these conditions, decarboxylative thioarylation with various thiol derivatives was performed, yielding the desired sulfide products with satisfactory yield. The results are shown in table 3.

**Table 3.** Synthesis of thioethers from the reaction of thiols and carboxylic acids in the presence of sodium thiosulfate^a^^,^^b^.

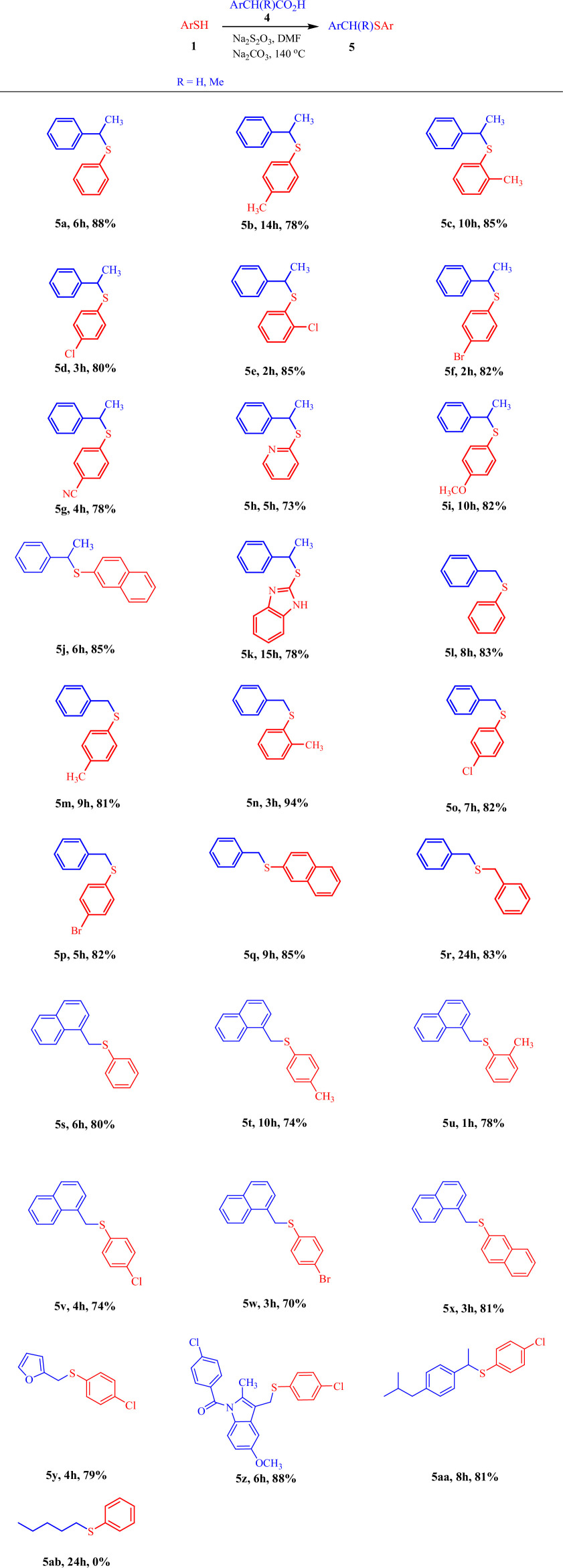		

^a^ Reaction conditions: thiol (1.0 mmol), carboxylic acid (1.0 mmol), Na_2_S_2_O_3_ (0.2 mmol), Na_2_CO_3_ (2.0 mmol) in DMF (1 ml) at 140°C.

^b^ The yields refer to the isolated pure products.

As shown by the data in the table 3, the reaction of 2-phenylpropanoic acid with thiophenol and its derivatives, pyridin-2-thiol, naphthalene-2-thiol and 1*H*-benzo[*d*]imidazole-2-thiol proceeded smoothly in accordance with the optimized reaction conditions, affording the corresponding sulfide products in acceptable yields (table 3, compounds 5a–5k). To our delight, when phenylacetic acid (table 3, compounds 5l–5r), 1-naphthaleneacetic acid (table 3, compounds 5s–5x) or 2-furanacetic acid (table 3, compound 5y) were used as substitutes for 2-phenylpropanoic acid, the reaction proceeded smoothly and give the corresponding adduct in high yields. As expected, hexanoic acid did not produce the desired product under the optimized conditions (table 3, compound 5ab).

To further evaluate the applicability of this method to more complex and biologically relevant substrates, we applied the optimized reaction conditions to two commercially available non-steroidal anti-inflammatory drugs (NSAIDs), indomethacin and ibuprofen, which both contain carboxylic acid functional groups. Gratifyingly, under our catalytic system, these drug-derived acids underwent smooth decarboxylative thioetherification with thiols to afford the corresponding thioether products (table 3, compounds 5z and 5aa) in acceptable yields. This successful transformation highlights the excellent functional group tolerance of the present method and its potential utility for late-stage functionalization of drug-like molecules. The ability to directly modify pharmacologically active compounds under mild and metal-free conditions further demonstrates the practical and environmentally benign nature of this approach.

Since these reactions are accompanied by the elimination of CO or CO_2_, it seems that the reaction must follow a radical pathway. To obtain further information about the reaction mechanism, a radical inhibitor was used in the model reaction, and two factors, time and yield, were examined. The results showed that in the presence of 2,2,6,6-tetramethylpiperidin-1-yl)oxyl (TEMPO) as a radical scavenger, the reaction was halted, and the yield of the sulfide product significantly decreased. Therefore, the reactions most likely proceed via a radical pathway, leading to thioylation coupled with decarbonylation/decarboxylation of aldehydes/carboxylic acids.

**Scheme 2.**
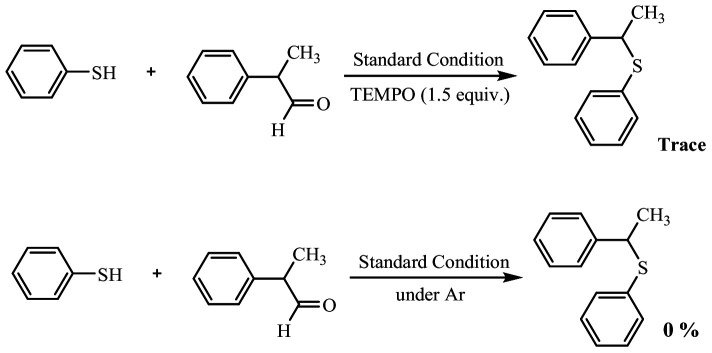
Control experiments.

To demonstrate the necessity of oxygen in the above reactions, another control reaction was conducted. For this purpose, the model reaction was performed in the absence of oxygen under an argon atmosphere. Under these conditions, no product was formed (Scheme 2). These findings suggest that the reaction mechanism is most likely radical in nature, with oxygen playing a crucial and important role in this mechanism.

**Scheme 3.**
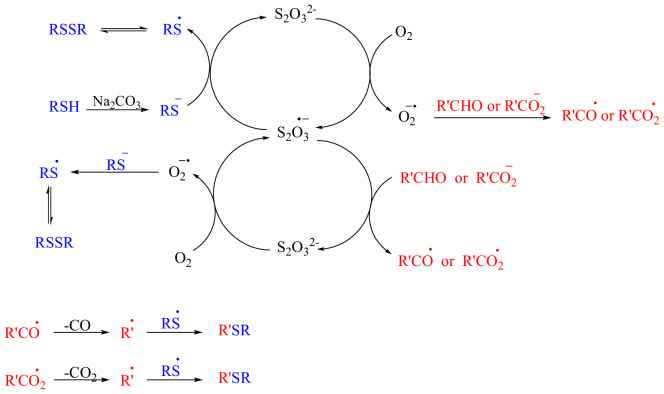
Proposed mechanism for the synthesis of thioethers.

Although the exact mechanistic role of thiosulfate in the reaction is not fully understood, based on the aforementioned results and control experiments, a possible mechanism is proposed in Scheme 3.

The catalytic cycle begins with the reaction of thiosulfate (S₂O₃²⁻) with molecular oxygen (O₂), leading to the formation of the thiosulfate radical anion (S₂O₃⁻^•^). This radical intermediate either reacts with an aldehyde (carboxylic acid) to produce an acyl radical (acyloxy radical) or interacts with the thiolate ion, which forms through the deprotonation of a thiol by sodium carbonate, to yield a thiyl radical. These reactions regenerate the thiosulfate ion, ensuring the catalytic cycle continues. In parallel, molecular oxygen (O₂) is also converted into a superoxide radical anion (O₂⁻^•^), which can mimic the behaviour of the thiosulfate radical anion (S₂O₃⁻^•^). The superoxide radical participates in generating the acyl radical R'CO^•^ (acyloxy radical R'CO_2_^•^) by reacting with the aldehyde (carboxylic acid) and can also interact with the thiolate (RS⁻) to form the thiyl radical (RS^•^). Next, it is reduced to hydrogen peroxide (H₂O₂), completing its role in the reaction mechanism. The generated hydrogen peroxide can facilitate the reaction by abstracting hydrogen atoms from thiols alongside sodium thiosulfate and is ultimately converted to water. Finally, the acyl radical (acyloxy radical) undergoes decarbonylation (decarboxylation), losing its carbonyl group (carboxyl group) to produce an alkyl radical (R'^•^), which then reacts with the thiyl radical (RS^•^), leading to the formation of the desired sulfide product (R'SR).

**Scheme 4.**
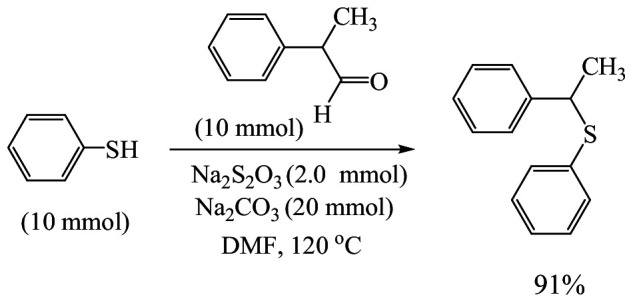
Large-scale synthesis.

To demonstrate the high plausibility and productivity of the present system, a scale-up reaction of thiophenol (10 mmol, 1 ml) and 2-phenylpropanal (10 mmol, 0.7 ml) was tested. As shown in Scheme 4, phenyl(1-phenylethyl)sulfane was effectively obtained in 91% yield.

## 3. Experimental

### 3.1. General

Thiols, aldehyde and carboxylic acids, sodium thiosulfate and sodium carbonate were commercially accessible. All additional reagents and solvents were purchased commercially and purified according to standard procedures. Column chromatography was accomplished on small columns of silica gel. ^1^H nuclear magnetic resonance (NMR; 300 and 400 MHz) and ^13^C NMR (75 and 100 MHz) spectra were run on Bruker in pure deuterated chloroform. Chemical shifts are given in the δ scale in parts per million (ppm) and *J* in Hz. Data for ^1^H NMR are reported as follows: chemical shift (δ ppm), multiplicity (s = singlet, d = doublet, t = triplet, q = quartet, m = multiple, dd = doublet of doublets), coupling constant (Hz) and integration.

### 3.2. General procedure for the synthesis of thioethers

A flask containing DMF (1 ml) equipped with a magnetic stirring bar was charged with thiol (1.0 mmol), aldehyde or carboxylic acid (1.0 mmol), Na_2_S_2_O_3_ (0.2 mmol) and Na_2_CO_3_ (2.0 mmol). This mixture was stirred at 120°C for aldehydes and 140°C for carboxylic acids in an oil bath. After completion of the reaction, which was detected by thin layer chromatography, the resulting solution was cooled down to room temperature, diluted with H_2_O (1 ml) and extracted with ethyl acetate (4 × 1 ml). The solvent was evaporated and the residue was purified by column chromatography on silica-gel using *n*-hexane as eluent to provide with the desired sulfide product.

## 4. Conclusion

In conclusion, in this study, a simple, efficient and environmentally friendly method for the synthesis of thioethers from aldehydes and carboxylic acids was developed using sodium thiosulfate as a catalyst. This radical-based method enables the formation of carbon–sulfur (C–S) bonds through decarbonylation or decarboxylation steps, achieving high yields. The results demonstrate that sodium thiosulfate, as an inexpensive and effective catalyst, facilitates coupling reactions between aldehydes or carboxylic acids and thiols under metal-free conditions, with DMF as the optimal solvent. Various functional groups on the thiols were well-tolerated under the reaction conditions, and the corresponding products were obtained with good to excellent yields, highlighting the broad substrate scope of this methodology.

To the best of our knowledge, this is only the second reported method to synthesize thioethers directly from aldehydes, and the number of studies in this area remains very limited. Additionally, the scalability of this method was demonstrated, as it successfully produced the desired product in high yield on a large scale. This highlights its practical applicability for industrial and academic purposes.

Importantly, the method was also successfully applied to complex, bioactive molecules such as the commercially available NSAIDs indomethacin and ibuprofen, both of which contain carboxylic acid groups. Their smooth transformation under mild, metal-free conditions underscores the excellent functional group tolerance of the system and demonstrates its potential for late-stage functionalization of drug-like molecules in an environmentally benign manner.

## Data Availability

Supplementary material contains spectral data and copies of ^1^H NMR and ^13^C NMR of the products [30].
